# A new mutation of congenital methemoglobinemia exacerbated after methylene blue treatment

**DOI:** 10.1002/ams2.335

**Published:** 2018-02-15

**Authors:** Fuminori Yamaji, Akio Soeda, Hiroki Shibata, Takuya Morikawa, Kodai Suzuki, Shozo Yoshida, Shinji Ogura

**Affiliations:** ^1^ Department of Emergency and Disaster Medicine Gifu University Graduate School of Medicine Gifu Japan; ^2^ Medical Institute of Bioregulation Division of Genomics Kyushu University Fukuoka Japan

**Keywords:** Ascorbic acid, congenital methemoglobinemia, CYB5R3, riboflavin

## Abstract

**Case:**

Methylene blue is useful for the treatment of methemoglobinemia. However, even after the patient's methemoglobin (metHb) rate has improved, careful observation is important because they could have undiagnosed congenital methemoglobinemia. In this case, a 67‐year‐old man underwent gastrointestinal endoscopy with the use of lidocaine for local anesthesia. During the examination, he complained of dyspnea and had low SpO_2_ despite normal PaO_2_ and SaO_2_. He was transferred to our department as a suspected case of acquired methemoglobinemia.

**Outcome:**

The patient's metHb level was 26.2%. We administered methylene blue i.v. and his metHb level subsequently decreased to 1.6%. However, his metHb level gradually increased to 18.2%, and we suspected that he had congenital methemoglobinemia. We administered riboflavin and ascorbic acid orally, and his metHb level decreased to 6.4%. We also obtained genomic DNA from the patient and identified a novel variant of *CYB5R3*.

**Conclusion:**

We report a novel variant of congenital methemoglobinemia that deteriorated after methylene blue treatment.

## Introduction

Methemoglobinemia is a rare condition and classified as either acquired or congenital. Acquired methemoglobinemia is more common and results from exposure to several oxidizing agents such as dapsone and acetaminophen as well as local anesthetics.^1^ Methemoglobin (metHb) levels >10% cause clinically recognizable cyanosis, >30% cause confusion, and >70% is lethal.^2^ Both the recessive and dominant forms of congenital methemoglobinemia have been reported and are characterized by diminished enzymatic reduction of metHb. These patients have abnormal amounts of metHb and low oxygen‐carrying capacity, and their metHb levels may be easily elevated by several oxidizing agents. Oxygenation and i.v. methylene blue are effective for the treatment of both acquired and congenital forms. Although this treatment rapidly reduces metHb levels, the levels can increase again to their daily levels in patients with congenital methemoglobinemia.

Here, we present the first case of congenital methemoglobinemia with a novel variant in exon 5 of *CYB5R3*. The metHb levels in this patient were elevated with local anesthesia and decreased with subsequent treatments with methylene blue; nevertheless, his metHb levels increased over 15%. Vitamins successfully suppressed hereditary metHb over a 1‐year follow‐up.

## Case

A 67‐YEAR‐OLD MAN had a loss of appetite, which prompted him to visit his primary care physician. He underwent gastrointestinal endoscopy with the use of 8% lidocaine spray for local anesthesia. During an examination, he complained of dyspnea and had low SpO_2_ (88% on 100% oxygen). However, his arterial blood gases showed normal PaO_2_ and SaO_2_ (PaO_2_, 206 mmHg; SaO_2_, 99.0%). Therefore, he was transferred to our department as a suspected case of acquired methemoglobinemia.

On his arrival, his metHb level was 26.2%; hence, we immediately administered 1 mg/kg methylene blue i.v. over 5 min in an established manner. One hour later, his metHb level dropped to 5.2% and his dyspnea and cyanosis was reduced. His metHb level decreased to 1.6% the next day. However, his metHb levels then gradually increased to 3.4% on day 3, 11.9% on day 8, and 18.2% on day 15. Therefore, we suspected that he had type I congenital methemoglobinemia. He also had cyanosis but not dyspnea, and increases in metHb levels may cause a recurrence of dyspnea. We then administered riboflavin (60 mg per day) and ascorbic acid (600 mg per day) orally, and his metHb level decreased to 15.8% after 3 days and dramatically decreased to 6.4% after 1 week. He was then discharged from our hospital without any complications, and also maintained the low metHb level (5.6%).

*CYB5R3* is known to be a major gene responsible for congenital metHb.^3^ We obtained genomic DNA from the patient and his healthy sister with approval from the Ethical Medical Review Board of Gifu University (Gifu, Japan) and the Ethics Committee of the School of Medicine, Kyushu University (Fukuoka, Japan). All exons of *CYB5R3* were examined by Sanger sequencing.^4^ The patient was homozygous for one single nucleotide variant (SNV), c.402G>C (Met134Ile), in exon 5, whereas his sister was GG homozygous for the SNV. We did not observe any other sequence variants for the other eight exons examined in *CYB5R3*. The amino acid substitution, Met134Ile, was predicted to be benign by PolyPhen2 (http://genetics.bwh.harvard.edu/pph2/) but damaging by SIFT (http://sift.jcvi.org/). We also examined the frequency of this SNV in the public databases. There were no records of this variant in public databases of human nucleotide variants, such as ExAC (http://exac.broadinstitute.org/) or ToMMo (https://ijgvd.megabank.tohoku.ac.jp/), indicating that this variant is absent in the healthy population.

## Discussion

Most congenital methemoglobinemias are caused by a cytochrome b_5_ reductase deficiency. This deficiency can be of two types: type I (also called erythrocyte reductase deficiency) occurs when red blood cells lack the enzyme, and type II (also called generalized reductase deficiency) occurs when the function of this enzyme is altered. In type II deficiency, cyanosis is accompanied by neurological impairment and reduced life expectancy.^5^ However, in type I, clinical symptoms are generally insignificant even with metHb levels up to 40%.^6^ Thus, patients with type I deficiency are able to have a normal life without any limitations in their daily activities. However, these patients are sensitive to oxidants, and reducing their metHb levels is useful in emergency cases.

Intravenous methylene blue is the primary treatment for decreasing metHb levels by activating nicotinamide adenine dinucleotide phosphate–metHb reductase in hemoglobin through the non‐enzymatic pathway.^7^ Conversely, ascorbic acid and riboflavin are effective for the treatment of congenital methemoglobinemia.^8^, ^9^ Ascorbic acid reduces excessive oxidative stress, and riboflavin can accelerate the reduction of methemoglobin levels through the nicotinamide adenine dinucleotide–flavin reductase system.^9^ These vitamins are effective for increasing metHb levels in congenital methemoglobinemia after methylene blue treatment.

## Conclusion

We have to keep in mind that patients who are admitted to the hospital with a suspected case of acquired methemoglobinemia may instead have congenital methemoglobinemia. In cases of congenital methemoglobinemia, metHb levels may increase after methylene blue treatment; therefore, careful observation is mandatory. Ascorbic acid and riboflavin are useful for alleviating hereditary methemoglobinemia.

## Disclosure

Approval of the research protocol: The study design was approved by the appropriate ethics review boards. We obtained genomic DNA from participants with approval from the Ethical Medical Review Board of Gifu University and the Ethics Committee of the School of Medicine, Kyushu University.Informed consent: All study participants provided informed consent. Registry and registration no. of the study/trial: N/A. Animal studies: N/A.Conflict of interest: None declared.

## References

[1] Nascimento TS , Pereira RO , de Mello HL , Costa J . Methemoglobinemia: from diagnosis to treatment. Rev. Bras. Anestesiol. 2008; 58: 651–64. 19082413 10.1590/s0034-70942008000600011

[2] Daly JS , Hultquist DE , Ruckenagel DL . Phenazopyridine induced methaemoglobinaemia associated with decreased activity erythrocyte cytochrome b_5_ reductase. JAMA 1951; 146: 24–5. 10.1136/jmg.20.4.307 PMC1049126 6620333

[3] Yubisui T , Murakami K , Shirabe K *et al* Structural analysis of NADH‐cytochrome b 5 reductase in relation to hereditary methemoglobinemia. Prog. Clin. Biol. Res. 1989; 319: 107–21. 2695933

[4] Miura S , Morikawa T , Fujioka R *et al* A novel frameshift mutation of DDHD1 in a Japanese patient with autosomal recessive spastic paraplegia. Eur. J. Med. Genet. 2016; 59: 413–6. 27216551 10.1016/j.ejmg.2016.05.010

[5] Percy MJ , Lappin TR . Recessive congenitall methaemoglobinaemia: cytochrome b_5_ reductase deficiency. Br. J. Haematol. 2008; 141: 298–308. 18318771 10.1111/j.1365-2141.2008.07017.x

[6] Jaffe E . Hereditary methemoglobinemias associated with abnormalities in the metabolism of erythrocytes. Am. J. Med. 1962; 32: 512. 10.1016/0002-9343(66)90037-4 5332175

[7] Cortazzo JA , Lichtman AD . Methemoglobinemia: a review and recommendations for management. J. Cardiothorac. Vasc. Anesth. 2014 Aug; 28: 1043–7. 23953868 10.1053/j.jvca.2013.02.005

[8] Park SY , Lee KW , Kang TS . High‐dose vitamin C management in dapsone‐induced methemoglobinemia. Am. J. Emerg. Med. 2014; 32: 684.e1–3. 10.1016/j.ajem.2013.11.036 24439259

[9] Yubisui T , Takeshita M , Yoneyama Y . Reduction of methemoglobin through flavin at the physiological concentration by NADPH‐flavin reductase of human erythrocytes. J. Biochem. 1980; 87: 1715–20. 7400118 10.1093/oxfordjournals.jbchem.a132915

